# The role of Neuropilin-1 in COVID-19

**DOI:** 10.1371/journal.ppat.1009153

**Published:** 2021-01-04

**Authors:** Bindu S. Mayi, Jillian A. Leibowitz, Arden T. Woods, Katherine A. Ammon, Alphonse E. Liu, Aarti Raja

**Affiliations:** 1 Department of Basic Sciences, Dr. Kiran C. Patel College of Osteopathic Medicine, Nova Southeastern University, Clearwater, Florida, United States of America; 2 Dr. Kiran C. Patel College of Osteopathic Medicine, Fort Lauderdale, Florida, United States of America; 3 USF Morsani College of Medicine, Tampa, Florida, United States of America; 4 Department of Biological Sciences, Halmos College of Arts and Sciences, Nova Southeastern University, Fort Lauderdale, Florida, United States of America; Boston University, UNITED STATES

## Abstract

Neuropilin-1 (NRP-1), a member of a family of signaling proteins, was shown to serve as an entry factor and potentiate SARS Coronavirus 2 (SARS-CoV-2) infectivity in vitro. This cell surface receptor with its disseminated expression is important in angiogenesis, tumor progression, viral entry, axonal guidance, and immune function. NRP-1 is implicated in several aspects of a SARS-CoV-2 infection including possible spread through the olfactory bulb and into the central nervous system and increased NRP-1 RNA expression in lungs of severe Coronavirus Disease 2019 (COVID-19). Up-regulation of NRP-1 protein in diabetic kidney cells hint at its importance in a population at risk of severe COVID-19. Involvement of NRP-1 in immune function is compelling, given the role of an exaggerated immune response in disease severity and deaths due to COVID-19. NRP-1 has been suggested to be an immune checkpoint of T cell memory. It is unknown whether involvement and up-regulation of NRP-1 in COVID-19 may translate into disease outcome and long-term consequences, including possible immune dysfunction. It is prudent to further research NRP-1 and its possibility of serving as a therapeutic target in SARS-CoV-2 infections. We anticipate that widespread expression, abundance in the respiratory and olfactory epithelium, and the functionalities of NRP-1 factor into the multiple systemic effects of COVID-19 and challenges we face in management of disease and potential long-term sequelae.

## Introduction

Neuropilin-1 (NRP-1), a member of a family of signaling and catalytic proteins, was recently shown to serve as an entry factor and potentiate SARS Coronavirus 2 (SARS-CoV-2) infectivity in vitro, although it is unclear whether NRP-1 enables attachment and receptor-mediated endocytosis in infected patients [1,2]. NRP-1 is most widely known for its role in cellular signaling and its function as a cell surface receptor [3]. Neuropilins are unique to vertebrates, and so far, zebra fish, frog, chick, mouse, rat, and human NRP homologues have been partially or completely identified, with expression patterns varying across species [4]. NRP-1 has 2 isoforms: a truncated, secreted form, and a transmembrane form which interacts with SARS-CoV-2 [1,5]. The secreted form of NRP-1 (sNRP-1) has been known to inhibit cell associated NRP-1 function in cervical cancer, with circulating sNRP-1 levels in patients with cervical cancer significantly higher than those of controls [6]. Knockdown of NRP-1 or pretreatment of Epstein–Barr virus (EBV) with soluble NRP-1 suppresses EBV infection of human nasopharyngeal epithelial cells, while up-regulation of NRP-1 by overexpression enhances EBV infection [7]. The transmembrane form of NRP-1 also has the ligand binding site, normally intended for growth factors such as vascular endothelial growth factor (VEGF), but now co-opted by viruses like EBV, human T-cell lymphotropic virus-1 (HTLV-1), and SARS-CoV-2 [1,2,7–9].

Upon infection, the SARS-CoV-2 Spike (S) protein is cleaved by host cell protease, furin, into S1 and S2 polypeptides, thereby exposing the CendR motif in S1 [2]. This motif is named for the “C-end terminal rule,” which is the requirement for the presence of a cationic amino acid, usually arginine, at the carboxyl terminus of the ligand, resulting in an RXXR configuration [10]. The CendR binding pocket lies within the b1 domain of NRP-1 [10]. Daly and colleagues [2] recently showed that the CendR motif in SARS-CoV-2 S1 protein binds to NRP-1 and potentiates virus infectivity. A monoclonal antibody against the extracellular CendR binding pocket in the b1 domain of NRP-1 reduces SARS-CoV-2 infectivity [2]. Analysis of NRP-1 RNA expression in cells isolated from bronchioalveolar lavage of patients with severe Coronavirus Disease 2019 (COVID-19) showed elevated expression in SARS-CoV-2–positive cells, but not uninfected cells [1]. It remains to be seen whether blocking the interaction between SARS-CoV-2 and NRP-1 could be an effective therapy in the fight against COVID-19.

Cantuti-Castelvetri and colleagues [1] also analyzed NRP-1 protein expression using the Human Protein Atlas [11], revealing high NRP-1 expression in the epithelial surface layer of the respiratory and gastrointestinal tracts, both of which are known to be affected by COVID-19 [12]. Although NRP-1 is disseminated throughout the tissues of the body, it is most commonly expressed by blood endothelial cells, vascular smooth muscle cells, and mesenchymal stem cells [13]. NRP-1 can also be expressed by adipose tissue macrophages, pulmonary and vascular endothelial cells, retinal vasculature, neurons, as well as immune cells such as CD8^+^ T cells, T-regulatory cells, and alveolar, bronchial, and vascular macrophages [14–17]. NRP-1 acts as a receptor for signaling ligands such as semaphorins, VEGF (specifically the VEGF-A isoform), transforming growth factor beta (TGF-β), plexins, and integrins [3,18,19]. NRP-1 plays a multisystem role in angiogenesis, tumor progression, viral entry, axonal guidance within the central and peripheral nervous systems, and immune function [16]. The diverse expression and functionality of NRP-1 makes it an ideal extracellular target for SARS-CoV-2 and may contribute to the multisystem impact of this viral infection.

NRP-1 interactions with VEGF and semaphorins have been shown to promote the growth, survival, and self-renewal of tumors [19]. It is expressed in a variety of different cancers, including glioblastomas, medulloblastomas, and carcinomas of the breast, prostate, and pancreas [3,19,20]. Given its widespread presence and role in tumorigenesis, NRP-1 has been the target of antitumor drugs, with peptide-based inhibition of NRP-1 leading to antiangiogenesis, and inhibition of tumor cell proliferation and migration [20].

### Vascular expression and function

NRP-1 complexes with VEGF receptor 2 (VEGFR2) in vascular endothelial cells to induce vessel permeability and angiogenesis [15,21]. However, VEGFR2 does not complex directly with NRP-1 but forms a bridge between the 2 entities via VEGF165, a specific VEGF isoform, which binds at the b1 domain of NRP-1, the same domain shown to bind the SARS-CoV-2 spike protein [2,22]. Targeting the VEGF-A binding domain of the NRP-1/VEGFR2 complex provides therapeutic benefits for anti-neovascularization in diseases such as rheumatoid arthritis, inflammatory diseases, arterial and reperfusion injuries, and atherosclerosis [23]. Gain-of-function mutations in mouse NRP-1 showed excessive vascularization, vessel dilation and hemorrhage, as well as embryo fatality [13]. Loss-of-function mutation of NRP-1 also showed lethality, in addition to impairment of angiogenesis in the brain, yolk sac, central nervous system (CNS), retina, and subventricular vascular plexus of the mouse embryo [13]. Although VEGF cannot function without NRP-1, the reverse doesn’t seem to be true as NRP-1 can perform its angiogenic functions without VEGF [13]. Mice expressing an altered NRP-1, incapable of binding VEGF, still showed postnatal survival demonstrating proper cardiovascular development. This VEGF-independent function of NRP-1 occurs through modulation of endothelial cell adhesion by integrins, fibronectin, and laminin, and by the use of TGF-β as an alternate binding partner during vascular development [13].

As the VEGF dependence on NRP-1 implies, the latter protein is critical to the maintenance and functioning of the vascular system. NRP-1 is up-regulated in endothelial and vascular smooth muscle cells after arterial injury and in response to fibroblast growth factor and platelet-derived growth factor (PDGF) [13,24]. This up-regulation after injury also occurs in the context of iatrogenic procedures that might stress the vasculature, such as angioplasty and stenting [24]. Up-regulated NRP-1 was associated with poor procedure outcome, specifically restenosis, due to neointimal hyperplasia, an important clinical occurrence post vascular surgery, which has the potential to limit the effectiveness of these surgical interventions. When NRP-1 was inhibited, this problematic neointimal thickening and re-endothelialization was likewise decreased [24]. Targeting NRP-1 may provide a potential therapeutic benefit by enhancing anti-VEGF functions when treating specific tumors [20]. For instance, the experimental drug, pTM-NRP-1, showed therapeutic benefits as an antiangiogenic and antitumor agent in the treatment of gliomas [20]. The transmembrane domain of NRP-1 has been a useful target when treating gliomas, exhibiting anti-VEGF effects and offering therapeutic benefit for pathologies aiming to block angiogenesis and tumor progression [20].

Severe COVID-19 also causes arterial injury, with the resulting potential up-regulation of NRP-1 pointing to the possibility of targeting NRP-1 for treatment of COVID-19 [25]. As mentioned earlier, researchers have shown elevated NRP-1 RNA expression in SARS-CoV-2–positive cells, but not uninfected bystander cells, isolated from bronchioalveolar lavage of patients with severe COVID-19 [1]. In an analysis of cryopreserved human diabetic kidney single-nucleus RNA sequencing dataset, of the several proposed factors involved in the entry of cells by SARS-CoV-2 including angiotensin-converting enzyme 2 (ACE2), only NRP-1 was significantly up-regulated [1]. This could be one of the reasons for the increased risk of SARS-CoV-2 infections in patients with diabetes.

Severe COVID-19 is associated with vascular pathologies like increased coagulation and sepsis [26]. Coagulation is controlled by the release and exposure of various factors on the endothelium after arterial injury or inflammation, the latter of which is also modulated by NRP-1. By controlling the adhesion and permeability of endothelial cells, NRP-1 could very well play a role in the pathology of coagulation. In vitro inhibition of NRP-1 by a photosensitizing peptide resulted in malfunctioning tumor vasculature caused by vasoconstriction, release of tissue factor, and the formation of thrombi [27]. By binding and outcompeting the traditional angiogenic ligands at the b1 domain of NRP-1, SARS-CoV-2 could promote dysfunction of the vasculature and coagulation throughout the body.

### NRP-1 in autoimmune conditions

The multifaceted role of NRP-1 in the immune system has made it an attractive potential target for immunotherapies such as monoclonal antibodies for use against autoimmune conditions, tumors, and perhaps infectious diseases like COVID-19 [1,17]. NRP-1 plays a role in alteration of both angiogenic and inflammatory activity in multiple systems and its effects as a receptor span several autoimmune diseases [3]. For instance, NRP-1 was shown to be essential in the treatment of experimental autoimmune encephalomyelitis, an animal model of demyelinating autoimmune disease [28]. Tuftsin tetrapeptide stimulated phagocytic activity of microglia in an NRP-1–dependent manner, revealing the significance of this receptor as a target for treatment of demyelinating diseases [28]. Knockout mice lacking NRP-1 expression showed a statistically significant weight loss, progressive demyelination of the spinal cord, and an increased activation time of T-regulatory cells in comparison to wild-type controls.

In rheumatoid arthritis, synoviocytes and angiogenic proliferation lead to a pannus followed by chronic inflammation. In experimentally induced rodent arthritis, anti-NRP-1 peptides formed from plasmin-dependent cleavage of VEGF165 were found to inhibit adhesion and migration of synoviocytes, as well as capillary proliferation and endothelial cell migration. Anti-NRP-1 peptides prevented binding of antiapoptotic factor VEGF165 to NRP-1 on endothelial cells and synoviocytes, leading to increased apoptosis of synoviocytes by decreasing expression of p-ERK and Bcl-2, factors essential to the survival of synoviocytes. Inhibition of these proliferative factors was shown to decrease angiogenesis, contributing to further evidence of the role of NRP-1 in vascular growth and as a potential target in the treatment of proliferative diseases [29].

NRP-1 levels in sputum and bronchoalveolar lavage of asthmatic patients were found to be significantly elevated when compared to non-asthmatic control groups [30]. It has been proposed that targeting NRP-1 could be utilized in the management of allergic diseases [30]. Although the ubiquitous nature of NRP-1 involves many different systems and diseases, the common theme of angiogenesis and the modification of the inflammatory response remains constant. What is unknown at this time is whether the involvement and up-regulation of NRP-1 in COVID-19 may translate into long-term consequences with impaired angiogenesis and/or immune functions.

### Neuronal expression and function

NRP-1 is abundant in the respiratory and olfactory epithelium, which may explain infectivity of SARS-CoV-2 in these epithelia as well as the possible route of spread through the olfactory bulb and into the CNS [1].

NRP-1 plays a central role in axonal guidance and pruning, particularly through its interaction with Semaphorin-3A (SEMA3A), a protein seen widely in both the nervous system and the vasculature [16,31]. When compensatory neuronal pathways are formed subsequent to a spinal cord injury [16], plexins act as signal transducers along with NRP-1, which is up-regulated in the motor cortex following the spinal cord injury. Blocking NRP-1 leads to inhibited axonal pruning of compensatory neural circuits within the CNS further highlighting the critical role of NRP-1 in cellular signaling and its role in the recovery of motor function after spinal cord injuries [16]. This pruning is also critical for sensory function. When NRP-1 expression was limited in the cochlea of mice embryos, postnatal mice demonstrated disorganized neurons and progressively worsening hearing loss [31]. With regards to olfaction, NRP-1 has been implicated in Kallman syndrome, a congenital disease characterized by hypogonadism and anosmia. Dysregulation in axonal guidance, caused by a malfunction in NRP-1 interaction with SEMA3A, leads to the development of this syndrome [3,32,33]. This last example is the most intriguing in the context of SARS-CoV-2, given that COVID-19 has a marked association with anosmia.

### Anosmia

While anosmia, an olfactory dysfunction, is a symptom seen in many upper respiratory illnesses [34], its presentation is unique in SARS-CoV-2. Rather than presenting with nasal inflammation and rhinorrhea, anosmia in the context of SARS-CoV-2 can occur unaccompanied by other symptoms [35]. A satisfactory scientific explanation for this phenomenon does not currently exist.

Brann and colleagues hypothesize that SARS-CoV-2–related anosmia does not involve the neurons, since the ACE2 receptor is not found in neurons [36]. Another hypothesis is that SARS-CoV-2 damages the olfactory cells by using ACE2 for entry [37–39]. However, there is one problem with this: ACE2 is not highly expressed in olfactory cells [1,40]. ACE2 is present in the nasal passage, in cells such as oligodendrocytes, goblet cells, or epithelial cells, but it is not widespread. ACE2 also does not explain how SARS-CoV-2 can infiltrate olfactory neurons themselves [1]. A recently published study suggests that there may be ACE2-independent pathways for SARS-CoV-2 entry and infection of neurons [41]. Therefore, we propose an alternate mechanism: SARS-CoV-2 entry into olfactory cells and neurons is potentiated by NRP-1.

NRP-1 is highly expressed in the olfactory epithelium and bulb, far more than ACE2 [1]. Instead of being expressed in a limited number of supportive cells, NRP-1 is expressed in nearly every cell type in the nasal passages. NRP-1 is even expressed in olfactory neurons, giving SARS-CoV-2 a direct path to enter those cells and disrupt olfaction. This pathway was modeled in mice, which had peptides containing the CendR motif that SARS-CoV-2 uses to bind NRP-1 administered to their olfactory epithelia. Within only 6 hours, these virion-sized peptides were found in the neuronal cells of the olfactory bulb. Anosmia could even be a harbinger of another SARS-CoV-2 sequelae, i.e., the NRP-1-facilitated infiltration of this virus into the CNS. In autopsies of patients who succumbed to COVID-19, SARS-CoV-2 was found in NRP-1-positive olfactory cells in nasal passages, the olfactory bulb, and olfactory tracts in the brain [1]. In the aforementioned mice experiment, the CendR-containing peptides were not only found in neurons of the olfactory bulb but were also found in NRP-1-positive parenchyma of the brain. Although SARS-CoV-2 does not use the CendR motif to bind ACE2, it uses it to bind NRP-1.

The high expression of NRP-1 in a variety of olfactory cells and its importance in olfactory neuronal maintenance makes it a stronger candidate in the mechanism of anosmia, compared to ACE2 [1]. Although Kallman syndrome occurs from errors in fetal neuronal development, that same NRP-1-SEMA3A signaling pathway is still utilized in adult neuronal maintenance and regeneration [42], introducing vulnerability to a potential loss of smell.

## Discussion

In the fast-changing landscape of what is known about SARS-CoV-2 infection and COVID-19, it is prudent to reconcile the latest scientific breakthroughs with more effective therapies. One way to do this is by repurposing old drugs. For instance, the discovery of NRP-1 as an entry or potentiating factor paves the way for drugs that target NRP-1. However, this approach has to be considered in conjunction with the role of NRP-1 in immunosuppression. Autopsies reveal the role of inflammation in the organ damage seen in deaths due to COVID-19 [43]. Targeting NRP-1 will need to be carefully monitored because of the widespread tissue expression, involvement of multiple bodily systems, including the immune system, and its potential dose-dependent effects.
